# Current topics of functional links between primary cilia and cell cycle

**DOI:** 10.1186/s13630-015-0021-1

**Published:** 2015-12-29

**Authors:** Ichiro Izawa, Hidemasa Goto, Kousuke Kasahara, Masaki Inagaki

**Affiliations:** Division of Biochemistry, Aichi Cancer Center Research Institute, 1-1 Kanokoden, Chikusa-ku, Nagoya, 464-8681 Japan; Department of Cellular Oncology, Nagoya University Graduate School of Medicine, 65 Tsurumai-cho, Showa-ku, Nagoya, 466-8550 Japan; Department of Oncology, Graduate School of Pharmaceutical Sciences, Nagoya City University, Nagoya, Aichi 467-8603 Japan

**Keywords:** Primary cilia, Cell cycle, Ciliogenesis, Ubiquitin–proteasome system, Cancer

## Abstract

Primary cilia, microtubule-based sensory structures, orchestrate various critical signals during development and tissue homeostasis. In view of the rising interest into the reciprocal link between ciliogenesis and cell cycle, we discuss here several recent advances to understand the molecular link between the individual step of ciliogenesis and cell cycle control. At the onset of ciliogenesis (the transition from centrosome to basal body), distal appendage proteins have been established as components indispensable for the docking of vesicles at the mother centriole. In the initial step of axonemal extension, CP110, Ofd1, and trichoplein, key negative regulators of ciliogenesis, are found to be removed by a kinase-dependent mechanism, autophagy, and ubiquitin–proteasome system, respectively. Of note, their disposal functions as a restriction point to decide that the axonemal nucleation and extension begin. In the elongation step, Nde1, a negative regulator of ciliary length, is revealed to be ubiquitylated and degraded by CDK5-SCF^Fbw7^ in a cell cycle-dependent manner. With regard to ciliary length control, it has been uncovered in flagellar shortening of *Chlamydomonas* that cilia itself transmit a ciliary length signal to cytoplasm. At the ciliary resorption step upon cell cycle re-entry, cilia are found to be disassembled not only by Aurora A-HDAC6 pathway but also by Nek2-Kif24 and Plk1-Kif2A pathways through their microtubule-depolymerizing activity. On the other hand, it is becoming evident that the presence of primary cilia itself functions as a structural checkpoint for cell cycle re-entry. These data suggest that ciliogenesis and cell cycle intimately link each other, and further elucidation of these mechanisms will contribute to understanding the pathology of cilia-related disease including cancer and discovering targets of therapeutic interventions.

## Review

Primary cilia are non-motile microtubule-based organelles that function as cellular antennae that sense a wide variety of signals during development and tissue homeostasis [1–6]. They are composed of nine doublet microtubules, named axoneme, elongated directly from the distal end of the basal body (mother centriole) [7–9] and are surrounded by a membrane lipid bilayer that maintains a lipid and protein content different from that of the plasma membrane [10–12]. The boundary between the ciliary and other cell compartments is demarcated by the transition zone [10]. Defects in formation, maintenance, and function of cilia result in human pathological conditions, including kidney cysts, retinal degeneration, brain malformations, obesity, and diabetes, called ciliopathies [1, 2, 10]. In addition, ciliary defects are implicated in cancer, because loss of cilia is commonly associated with various types of cancer [13–20], and the kidney cysts in polycystic kidney disease are associated with increased cell proliferation and often also with a loss of cell polarity, two features commonly related to tumorigenesis [2, 21].

**Fig. 1:**
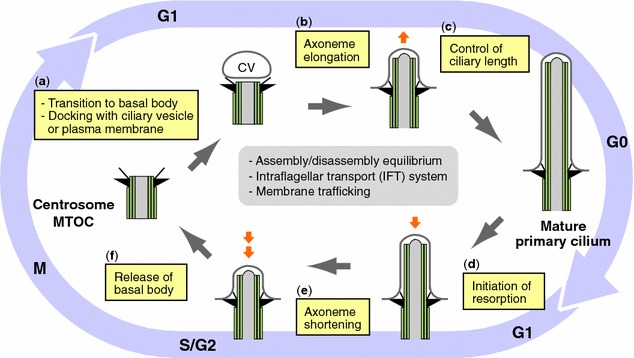
Ciliogenesis cycle and cell cycle. Multiple steps (**a**–**f**) of primary cilia formation in ciliogenesis cycle are shown in related to cell cycle

Ciliogenesis is a multi-step process that has been characterized in detail by ultra-structural examination of ciliated cells [22, 23]. Formation of primary cilia typically starts at the G1/G0 phase of the cell cycle and begins to disassemble as cells re-enter the cell cycle [24–26] (Fig. 1). Upon cell cycle exit, migration of the centrosome to the cell surface represents the first regulatory event of ciliogenesis, during which the mother centriole forms a basal body to nucleate ciliary axoneme [27] (Fig. 1a). Sorokin [28] described two physiologically relevant pathways to generate primary cilia, namely the extracellular and intracellular pathways [29, 30]. In the extracellular pathway, the mother centriole first docks to the plasma membrane after which axonemal microtubules are nucleated. In the intracellular pathway, the extension of the axoneme begins in the cytoplasm upon association of the mother centriole with vesicles, called the ciliary vesicles (CV), which are derived from the Golgi apparatus [31] (Fig. 1a). The axoneme assembly and elongation require the coordination of motor-driven intraflagellar transport (IFT), membrane trafficking, and selective import of cilium-specific proteins through a barrier at the ciliary transition zone [4, 32, 33] (Fig. 1b). The steady-state ciliary length is determined by the balance of ciliary assembly and disassembly [4] and it has been recently revealed that a cilium length control signal can regulate IFT cargo loading [34] (Fig. 1c). Upon cell cycle re-entry, ciliary resorption begins (Fig. 1d), and the balance of cilium assembly and disassembly is shifted toward disassembly [35] (Fig. 1e). Ciliary resorption has been most extensively studied in cell culture, where cells are arrested in G0 by serum starvation to form cilia and then are induced to re-enter the cell cycle using serum or defined growth factors [24, 26, 36]. After serum stimulation, the disassembly occurred in two waves, with the first occurring 1–2 h after serum stimulation and the second after 18–24 h in human RPE1 (telomerase reverse transcriptase-immortalized retinal pigment epithelial) cell line [25, 37]. Finally, the basal body is released from cilia, thereby freeing up centrioles (centrosome) to function as microtubule organizing center (MTOC) or spindle poles during mitosis [9, 27] (Fig. 1f).

**Fig. 2:**
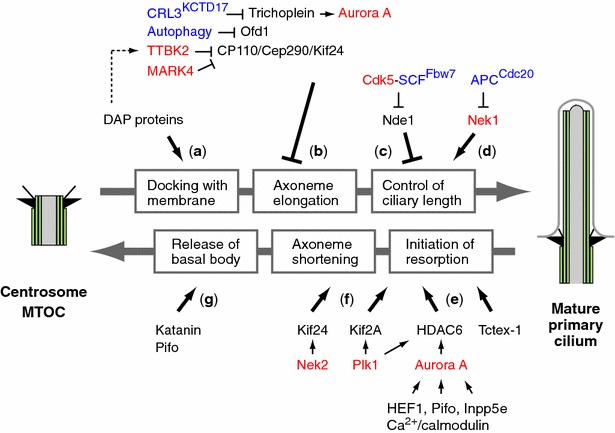
Molecular mechanisms linking ciliogenesis and cell cycle regulation. Recent findings to uncover the molecular link between the individual step (**a**–**g**) of ciliogenesis and cell cycle are depicted. *Red letters* indicate kinases and *blue letters* indicate molecules related to ubiquitin–proteasome pathway or autophagy

As the molecular composition of cilia assembly/disassembly system has been well defined in recent years, mechanisms how ciliogenesis and cell cycle progression are linked each other have attracted considerable attention. Since a number of comprehensive and excellent review articles on these issues have been published [8, 9, 13, 17, 26, 27, 38–40], we discuss here mainly the recent progress that provides clues to understand the linkage between the individual process of ciliogenesis cycle and cell cycle regulation (Figs. 1, 2).

## Regulation of the initiation of ciliogenesis by distal appendage (DAP) proteins

The distal appendage (DAP) proteins of the mother centriole have been found to be involved in membrane docking at the initial step of ciliogenesis [41, 42] (Fig. 2a). Nigg and colleagues first identified centrosomal protein 164 (Cep164) as an excellent marker for DAP, which is required for primary cilium formation [43]. Pereira and coworkers revealed that Cep164 is indispensable for the docking of vesicles at the mother centriole [41]. They identified the components of the vesicular machinery, the GEF Rabin8 and the GTPase Rab8, as interacting partners of Cep164, indicating that Cep164 is directly involved in recruiting Rabin8 to promote the local activation of Rab8 at the centrosome [41]. In addition, they found that Cep164 associates with centrosome in a cell cycle-dependent manner, as Cep164 protein levels at the centrosome decrease during mitosis to peak at this location in interphase of cycling or serum-starved cells [41], which appears to be inconsistent with a previous report [43]. It is also reported that knockdown of Cep164 accelerates the cell cycle but inhibits overall proliferation, because of its association with DNA damage-induced replicative stress, apoptosis, and epithelial-to-mesenchymal transition, representing the pathological mechanism of nephronophthisis, a polycystic kidney disease (PKD) [44]. A subsequent study identified five DAP components, including Cep164, Cep89 (CCDC123), Cep83 (CCDC41), SCLT1, and FBF1/Albatross, and revealed a hierarchy of DAP assembly [42]. Loss of Cep83, positioned at the top of the hierarchy, blocks centriole-to-membrane docking, and undocked centrioles fail to recruit TTBK2 or release CP110, the two earliest modifications found on centrioles prior to cilia assembly [42] (Fig. 2a, b). CCDC41/Cep83 also plays an important role in the recruitment of IFT20 to the basal body [45]. These data suggest that centriole-to-membrane docking mediated by DAP may serve as an instructive signal that temporally and spatially regulates cilia initiation [42].

## Ciliary extension triggered by destruction or dislocation of negative regulators of ciliogenesis

Some negative modulators of ciliogenesis have been recently found to be destructed or dislocated from ciliary structures at the onset of ciliogenesis [46–49], indicating that the removal of these proteins from the mother centriole functions as a restriction point to decide whether axoneme nucleation and extension starts or not (Fig. 2b).

### CP110 dislocation by TTBK2 and MARK4

CP110 is shown to localize to the distal ends of centrioles, forming a ‘cap’ above the growing microtubules that inhibits microtubule growth [50], suggesting that CP110 negatively regulates ciliary assembly. Kobayashi et al. demonstrated that Kif24, a kinesin-13 family protein with microtubule-depolymerizing activities, interacts with CP110 and Cep97 and preferentially localizes to mother centrioles [51]. They also observed that loss of Kif24 results in the disappearance of CP110 from mother centrioles, specifically in cycling cells able to form cilia, leading to unscheduled cilia formation but not promotion of abnormally long centrioles, unlike CP110 or Cep97 knockdown [51]. CP110 is also associates with Cep290 [52], a hub protein connecting a broad network of ciliary proteins [53], and Cep104 [54]. It seems that CP110 restrains Cep290 from promoting ciliogenesis at an early step of the ciliogenetic pathway in proliferating cells, but once cells exit the cell cycle, the loss of CP110 protein releases Cep290 from inhibition [53]. Two kinases, Tau tubulin kinase 2 (TTBK2) [46] and microtubule-associated protein/microtubule affinity regulating kinase 4 (MARK4) [47], are reported to initiate ciliogenesis by excluding CP110 from the mother centriole (Fig. 2b). Anderson’s group found that TTBK2, a spinocerebellar ataxia-associated protein, acts at the distal end of the basal body, where it promotes the removal of CP110 and facilitates the recruitment of IFT proteins, which build the ciliary axoneme [46]. As the recruitment of TTBK2 to the mother centriole in response to cell cycle signals immediately precedes the removal of CP110 from the mother centriole, TTBK2 may initiate ciliogenesis by phosphorylating one or more of the proteins in the CP110/Cep97/Cep290/Kif24 cilia-suppression pathway [46]. In addition, as described above, centriole-to-membrane docking mediated by DAP proteins is a prerequisite for the targeting of TTBK2 to the mother centriole and the removal of CP110 [42] (Fig. 2a, b). Pereira and colleagues observed the interaction of MARK4 and Odf2, a mother centriolar protein, and revealed that upon MARK4 or Odf2 knockdown, the ciliary program arrests before the complete removal of the CP110/Cep97 inhibitory complex from the mother centriole [47] (Fig. 2b). The precise molecular mechanisms of the removal of CP110 by TTBK2 and MARK4, including regulation of these processes during cell cycle by upstream signals and events, such as centriole-to-membrane docking, remain unclear at present. Clearly, the elucidation of these steps will lead to further understanding of early steps of ciliogenesis. CP110 also plays an essential role in centrosome duplication [50, 55, 56] and cytokinesis [57], and its expression levels and the localization to the centrosome are tightly regulated in a cell cycle-dependent manner, where CP110 protein levels drop significantly in G2/M and G0/G1 phases [55]. The tight control of CP110 levels during cell cycle is partly regulated through ubiquitination by Skp1/Cullin1/F-box protein (SCF) complexes SCF^cyclin F^ [58] and deubiquitination by USP33 [59], suggesting that the equilibrium between ubiquitination and deubiquitination governs the levels of a critical centrosome protein CP110 during the cell cycle, thereby preserving the fidelity of mitosis and genome integrity [59].

### Ofd1 removal through autophagy

Orofaciodigital syndrome 1/Oral-facial-digital syndrome 1 (Ofd1) acts at the distal centriole to build distal appendages, recruits IFT88, stabilizes centriolar microtubules at a defined length, and is required for primary cilia formation [60, 61]. Ofd1 also localizes to centriolar satellites, interacting with PCM1, Cep290, and BBS4 [62]. Zhong et al. [48] found that autophagic degradation of Ofd1 at centriolar satellites promotes primary cilia formation (Fig. 2b). Thus, Ofd1 at centriolar satellites has a crucial role in suppressing primary ciliogenesis, whereas Ofd1 at centrioles is essential for primary ciliogenesis [48].

### Trichoplein degradation by CRL3^KCTD17^

**Fig. 3:**
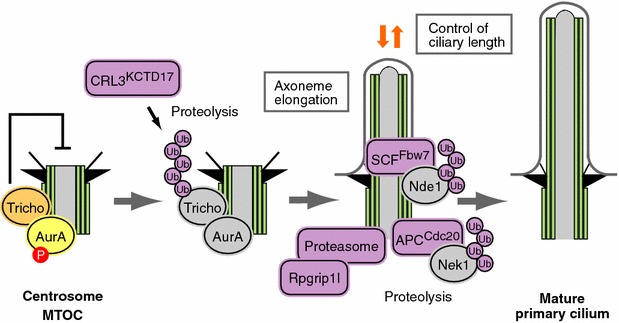
Involvement of the ubiquitin–proteasome system in ciliogenesis and cell cycle control. Ciliogenesis is regulated by the ubiquitin–proteasome system in a cell cycle-dependent manner

Trichoplein, originally identified as a keratin-binding protein [63], is concentrated at the subdistal/medial region of both mother and daughter centrioles and activates centriolar Aurora A kinase in growing cells [64]. During ciliogenesis, trichoplein disappears from the mother centrioles, and depletion of this protein in cycling RPE1 cells induces unscheduled primary cilia formation, whereas overexpression blocks ciliogenesis, indicating that trichoplein negatively controls ciliogenesis at the mother centrioles [64] (Fig. 2b). In proliferating RPE1 cells, trichoplein or Aurora A knockdown induced primary cilia formation, resulting in cell cycle arrest at the G0/G1 phase. This arrest can be reverted if primary cilia formation was blocked by simultaneously depleting IFT20 which is required for the assembly/maintenance of cilia and flagella [64–67], suggesting that primary cilia play an active role in blocking cell proliferation [38, 64]. Trichoplein also regulates the recruitment of microtubules to centrioles through interaction with Odf2 and ninein in non-ciliated HeLa cells [68]. Because trichoplein is concentrated at both centrioles in dividing cells and disappears specifically from the mother centriole/basal body [64], a mechanism regulating this removal of trichoplein from the mother centriole should exist. We have recently shown that ubiquitin–proteasome system removes trichoplein, a negative regulator of ciliogenesis, from the mother centrioles and thereby causes Aurora A inactivation, leading to ciliogenesis [49]. We have further identified KCTD17 as a substrate-adaptor for Cul3-RING E3 ligases (CRL3s) that polyubiquitinates trichoplein. Transmission electron micrographs of ciliogenesis in KCTD17-depleted cells revealed that KCTD17 is not required for the maturation of mother centriole and the centriole-to-membrane docking, but instead, plays a crucial role in the initial step of axoneme extension during ciliogenesis. Thus, CRL3^KCTD17^ targets trichoplein to proteolysis to initiate the axoneme extension during ciliogenesis [49] (Figs. 2b, 3). CRL3^KCTD17^ targets trichoplein to proteolysis in response to serum starvation, but the CRL3^KCTD17^ protein levels are unchanged. CRL3^KCTD17^ activity, therefore, may be modulated through posttranslational modification such as phosphorylation by TTBK2 or MARK4 [46, 47], or counteracted with an unidentified deubiquitylating enzyme like the case of CP110 [49, 58, 59].

## Ciliary length control and cell cycle

### *Nde1 regulation by CDK5*-*SCF*^*Fbw7*^

It is becoming clear that ciliary length can influence cell cycle time [17, 26]. Tsiokas and colleagues identified the mother centriolar protein Nde1 (nuclear distribution gene E homologue 1) as a negative regulator of ciliary length [65] (Figs. 2c, 3). Nde1 is expressed at high levels in mitosis and low levels in quiescence. Cells depleted of Nde1 have longer cilia and a delay in cell cycle re-entry that correlates with ciliary length [65]. Nde1 shortens ciliary length via its association with a dynein light chain protein, DYNLL1/LC8. Of note, they clearly showed that Nde1 affects cell cycle re-entry through cilia, because simultaneous knockdown of IFT88/Polaris or IFT20 suppresses cilia formation and reverses the effect of Nde1 depletion on the rate of cell cycle re-entry [65]. Furthermore, Tsiokas and colleagues have recently reported the fascinating finding that cell cycle-dependent mechanisms can control ciliary length through a CDK5-SCF^Fbw7^-Nde1 pathway [69], a precise molecular link between primary cilia and cell cycle (Figs. 2c, 3). They identified F-box and WD40 repeat domain-containing 7 (Fbw7) (also called Fbxw7, CDC4, AGO, or SEL-10) as the E3 ubiquitin ligase that mediates the destruction of Nde1 and maintains a low level of Nde1 during G1 and G0, allowing cilia to form and function properly. CDK5, a kinase active in G1/G0, phosphorylates and primes Nde1 for Fbw7-mediated recognition [69] (Figs. 2c, 3). Fbw7 is an F-box protein that is responsible for substrate recognition by an SCF-type ubiquitin ligase complex [70, 71]. SCF^Fbw7^ targets several proteins, including c-Myc, Notch1, c-Jun, and cyclin E, for degradation, and thereby functions as a tumor suppressor or is involved in the maintenance of normal stem cells and cancer-initiating cells [70, 71]. Thus, CDK5-SCF^Fbw7^-Nde1 pathway is not only implicated in the regulation of ciliary length by cell cycle but also critical to understand cilia biology in normal and cancer stem cells.

### Involvement of APC in ciliogenesis

Anaphase-promoting complex (APC), a key ubiquitin E3 ligase that controls the onset of anaphase, is reported to localize to the basal body, where it has a role in regulating ciliary polarity [72]. Kirschner and coworkers found that the activity of APC^Cdc20^ is required for maintaining the proper length of preformed cilia as well as for the timely resorption of the cilium after serum stimulation [73] (Figs. 2d, 3). They also found that APC^Cdc20^ regulates the stability of axonemal microtubules through targeting Nek1, a ciliary kinase, for ubiquitin-mediated proteolysis [73] (Figs. 2d, 3). As this result appears to be in sharp contrast to a report that BUBR1-dependent Cdc20 degradation in G0 phase cells plays a role in the maintenance of APC^CDH1^ activity which promotes the assembly of primary cilia [74, 75], further elucidation will be needed to resolve this issue.

### *Basal body*-*specific proteasomal machinery*

Trichoplein in CRL3^KCTD17^-trichoplein pathway, Nde1 in CDK5-SCF^Fbw7^-Nde1 pathway, and Nek1 in APC^Cdc20^-Nek1 pathway are all thought to be subjected to proteolysis at the basal body (Fig. 3). Gerhardt and colleagues have recently demonstrated that the ciliopathy protein Rpgrip1 l regulates proteasomal activity specifically at the basal body via interaction with Psmd2, a component of the regulatory proteasomal 19S subunit [76] (Fig. 3). Based on these results, ubiquitination and possibly deubiquitination of key regulators of ciliogenesis specifically at the basal body represent a major mechanism of controlling ciliogenesis. Besides ubiquitin E3 ligases described here, a subset of E3 ligases, including pVHL and MIB-1, are reported to be implicated in ciliogenesis [77–81].

### Ciliary length signal in *Chlamydomonas*

Elongation of cilia is dependent on delivery of structural components to their tips by IFT [4, 32]. Recent studies on ciliary length control indicates that during ciliary growth, cilia send an uncharacterized length signal to the cytoplasm that is converted into differential loading of cargo onto constitutively trafficking IFT complexes [34, 82–85]. In *Chlamydomonas*, ciliary length is controlled by genes encoding protein kinases, including the genes disrupted in several long flagella (LF) mutants, suggesting that length control is dependent on length signals sensed in cytosol [34, 83–85]. In addition, *Chlamydomonas* Aurora-like protein kinase (CALK) is phosphorylated at the activity-regulating residue Thr193 in the kinase activation loop, the phosphorylation state of which is dynamically related to flagellar length [82].

## Ciliary resorption upon cell cycle re-entry

### *Aurora A*-*HDAC6 pathway*

Aurora A kinase is a well-known kinase that regulates M phase entry and progression [86], and is also found to be a hub molecule to execute resorption of cilia (see for review [13, 26, 38, 39]) (Fig. 2e). Snell and coworkers first found that CALK controls resorption of the flagellum, an organelle similar to the mammalian cilium, during mating or in response to ionic stresses [87]. Golemis and colleagues subsequently showed that HEF1-dependent activation of Aurora A upon growth factor induced ciliary resorption in quiescent cells, and established that Aurora A is necessary and sufficient to induce cilium resorption [37] (Fig. 2e). They also demonstrated that activated Aurora A phosphorylates and activates histone deacetylase 6 (HDAC6), resulting in cilia disassembly [37]. Notably, the Aurora A activation by HEF1 is observed at 1–2 and ~18–24 h after serum stimulation, namely at each of the two waves of cilium disassembly, indicating that HEF1-Aurora A-HDAC6 pathway is a central component to disassemble cilia even during first (G1 resorption) wave of ciliary resorption [37] (Fig. 2e). Lickert et al. revealed that Pitchfork (Pifo), a mouse embryonic node protein, accumulates at the basal body during cilia disassembly and mediates Aurora A activation, inducing cilia retraction [88] (Fig. 2e). *Pifo* haploinsufficient mice show a unique node cilia duplication phenotype, left–right asymmetry defects, and heart failure [88], whereas *HEF1* knockout mice have only limited defects [38, 89], suggesting that the Aurora A activation in cilium disassembly can be redundantly regulated by several activators of Aurora A except during specific embryonic development. On the other hand, Golemis and coworkers found additional Aurora A activators, Ca^2+^ and calmodulin, in ciliary disassembly [90] (Fig. 2e). In addition, Plotnikova et al. have recently described the interaction of Aurora A and inositol polyphosphate 5-phosphatase E (Inpp5e), linking phosphoinositide signaling to primary cilium stability [91] (Fig. 2e). They showed that the reciprocal interaction between Aurora A and Inpp5e, including phosphorylation of Inpp5e by Aurora A, is important for the stability of primary cilia [91], through a mechanism that appears to be complex. Inpp5e is a lipid phosphatase localized exclusively at cilia where it removes the 5-phosphate group from PI(3,4,5)P3 and PI(4,5)P2 [92, 93]. Mutations in Inpp5e, described in patients with Joubert syndrome, accelerate ciliary disassembly, resulting in faster cell cycle re-entry [26, 92, 93]. Chávez et al. and Garcia-Gonzalo et al. have recently shown that Inpp5e keeps PI(4,5)P2 levels low or at a minimum to tightly control the trafficking of Hedgehog proteins and thereby regulates Hedgehog signaling at primary cilia [11, 94, 95].

### *Plk1*-*HDAC6 pathway*

It is reported that Polo-like kinase 1 (Plk1), a key cell cycle regulator, interacts with and activates HDAC6 to promote ciliary deacetylation and resorption before mitotic entry [96] (Fig. 2e). In this process, CDK1 first phosphorylates pericentriolar material 1 (PCM1), resulting in the recruitment of Plk1 to the pericentriolar matrix through the interaction between PCM1 and Plk1 [96]. Plk1 is also reported to stabilize HEF1, which enhances ciliary absorption by HEF1-Aurora A-HDAC6 pathway [97].

### *Tctex*-*1 in ciliary disassembly and cell cycle progression*

Sung and coworkers found that Tctex-1 phosphorylated at Thr 94 is recruited to ciliary transition zones before S phase entry and has a pivotal role in both ciliary disassembly and cell cycle progression, supporting a model in which cilia act as a brake to prevent cell cycle progression [98] (Fig. 2e). They also showed that Tctex-1 phosphorylated at Thr 94 has a key role in G1 length, cell cycle entry, and fate determination of cortical neuronal progenitor cells during corticogenesis [98]. Furthermore, Sung’s group found that insulin-like growth factor-1 (IGF-1) accelerates G1/S transition by causing cilia to resorb [36]. The mitogenic signals of IGF-1 are transduced via IGF-1 receptor (IGF-1R) on the cilia, and in turn phosphorylated IGF-1R activates an AGS3-regulated G_βγ_ signaling pathway that subsequently recruits phospho (Thr94) Tctex-1 to the transition zone [36]. During corticogenesis, a cilium-transduced IGF-1R- G_βγ_- phospho (Thr94) Tctex-1 pathway promotes the proliferation of neural progenitors through modulation of ciliary resorption and G1 length [36].

## Roles of microtubule-depolymerizing kinesins in ciliary resorption

The human kinesin-13 family proteins consist of Kif2A, Kif2B, Kif2C/MCAK, and Kif24, which have ATP-dependent microtubule-depolymerizing activity [99]. Miyamoto et al. have recently found that Kif2A, phosphorylated at Thr554 by Plk1, exhibits microtubule-depolymerizing activity at the mother centriole to disassemble the primary cilium coupled with cell proliferation [75] (Fig. 2f). They also described that Kif2A is degraded through the APC-mediated ubiquitin–proteasome system in the quiescent G0 phase. In Kif2A-deficient cells, primary cilia disassembly is inhibited 4 h after serum stimulation of quiescent cells, compared to control cells [75], indicating that the Plk1-Kif2A pathway works from the early phase of ciliary disassembly after serum re-stimulation (Fig. 2f).

Nek2, an S/G2 kinase, is reported to localize to the distal portion of the mother centriole and be required for timely cilium disassembly at the G2/M transition [100]. Dynlacht and coworkers have shown that Kif24, a kinesin-13 family protein, is phosphorylated by Nek2, which stimulates its microtubule-depolymerizing activity and prevents the outgrowth of cilia in proliferating cells [35] (Fig. 2f). They also suggested that cilium assembly and disassembly are in dynamic equilibrium, but Nek2 and Kif24 can shift the balance toward disassembly. It is noteworthy that Aurora A-HDAC6 and Nek2-Kif24 play distinct, sequential roles during cilia disassembly as cells re-enter the cell cycle from quiescence: Aurora A-HDAC6-mediated axonemal disassembly is succeeded by Nek2-Kif24-mediated suppression of nascent cilium assembly, and Kif24 activity could ensure the completion of cilium removal in the later stages of the cell cycle [35]. They further addressed that in breast cancer cells, aberrant activation of the Nek2-Kif24 pathway promotes cilium disassembly and proliferation, and abrogating this defective Nek2-Kif24 activation can restore primary cilia formation and restrict proliferation in breast cancer cells devoid of accumulated oncogenic hits [35]. Since Kif24 also interacts with CP110/Cep97 [51] as described earlier, Kif24 appears to orchestrate the early step of axonemal extension as well as the later stage and the completion of axonemal resorption.

## Two distinct phases in ciliary resorption

**Fig. 4:**
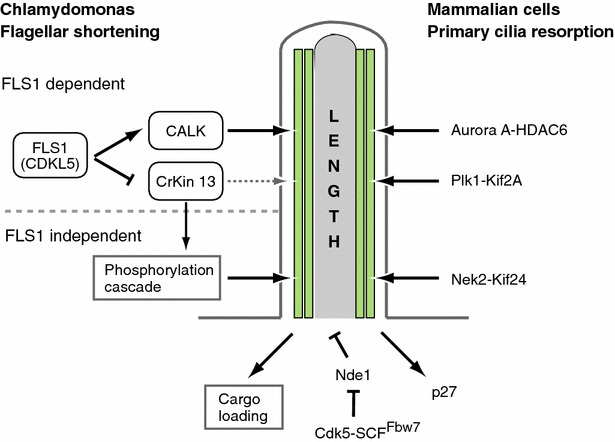
A model for cilia length and cell cycle. Molecular mechanisms of flagellar shortening of *Chlamydomonas* and primary cilia resorption in mammalian cells are shown (*upper* portion). In addition, a possible link between ciliary length signal and cell cycle is depicted (*lower* portion)

Together with the reports described above, it is currently plausible that ciliary resorption upon cell cycle re-entry in mammalian cells has two distinct phases: the first (G1 resorption) wave regulated mainly by Aurora A-HDAC6 and Plk1-Kif2A and the second (G2/M resorption) wave chiefly conducted by Nek2-Kif24 (Figs. 2e, f, 4). In mammalian cells, the first (distal) ciliary resorption is necessary for proper G1/S transition, whereas complete resorption is not [26, 98, 101, 102]. Thus, Pan and colleagues speculated that the first phase resorption may generate signals for S phase entry, and once the cell acquires the capacity to enter S phase, the second phase shortening would proceed, leading to resorption of the proximal portion of axoneme to release the basal body (centrosome) for mitotic spindle formation [98, 101, 103, 104]. In regard to this issue, Pan’s group has provided intriguing and suggestive findings in the flagellar shortening pathway of *Chlamydomonas,* supporting the speculation described above [101]. They revealed that flagellar resorption occurs in two distinct phases of length-dependent regulation, where a CDK-like kinase (CDKL5), encoded by *flagellar shortening* (*FLS1*), is required for the normal rate of disassembly of only the distal part of the flagellum [101] (Fig. 4). To exert this function, FLS1 induces the initial phosphorylation and activation of CALK that regulates flagellar shortening, and also inhibits the early phosphorylation of CrKinesin13, a microtubule depolymerase, the phosphorylation of which impairs its microtubule depolymerization activity in vitro [105]. Moreover, they found that ciliary shortening itself induces a phosphorylation cascade, revealing a mechanism in generation of ciliary signaling not requiring the binding of a ligand or the stimulation of an ion channel [101] (Fig. 4).

## Release of basal body

In *Chlamydomonas*, katanin, a microtubule-severing ATPase seems to serve to release basal bodies from their transition zones when flagella are resorbed, thus freeing basal bodies to migrate and facilitate proper placement of the spindle poles [106] (Fig. 2g). Pifo is also reported to be essential for releasing basal body from cilia and for ciliary retraction in mouse cells [27, 88] (Fig. 2g).

## Primary cilia function as a structural checkpoint for cell cycle re-entry

Ciliogenesis and cell division are thought to be mutually exclusive events as the centrioles must be released from the plasma membrane to function as a mitotic apparatus, albeit with some exceptions [9, 13, 27, 38]. Therefore, causal relationship between cilia and cell cycle was pointed out [8, 107], but their intimate interdependence has made it challenging to draw an unequivocal conclusion about it [27, 38, 104]. Several recent studies, however, have broken at least partially this deadlock, observing the effects of ciliary proteins on cell cycle progression in cilia-depleted condition by means of the knockdown of IFT88, IFT20, or Talpid3 [35, 64, 65, 98]. The data on trichoplein [64] and Nek2-Kif24 [35] proposed a model in which proper cell cycle progression requires continuous suppression of primary cilia formation in proliferating cells [38]. In addition, the works on Nde1 [65] and Tctex-1 [98] provided substantial supportive evidence for a concept that the presence of cilia itself can act as a brake or function as a physical checkpoint to prevent cell cycle re-entry [26]. From another point of view, these results might also indicate that the axonemal length directly influences cell cycle time [17]. That is, the abnormally longer cilia take more time to resorb and become ready for re-entry into the cell cycle than the normal cilia, and the reduction of cilia length or cilia-depletion allows cells to enter S phase more rapidly [17].

Because many tumors often lack cilia as described earlier, these models also suggest a straightforward therapeutic principle stating that the restoration of primary cilia in tumor cells could suppress tumor proliferation [38, 64]. Supporting this, Dynlacht and colleagues demonstrated that depletion of Nek2, a proto-oncogene that is highly expressed in various cancer types, or of Kif24 could rescue ciliogenesis and thereby restrict proliferation in breast cancer cells, although there is no impact of deletion in a most invasive cell line possibly due to the accumulation of genetic alterations [35].

**Fig. 5:**
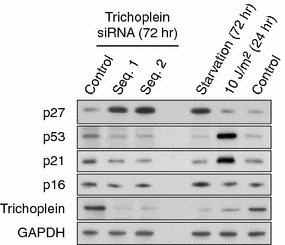
Effects of trichoplein depletion on cell cycle regulators. Proliferating RPE1 cells were transfected with siRNA for control or trichoplein (#1 and #2), and then cultured for 72 h. The cell extracts were subjected to immunoblotting analysis with p27^Kip1^, p53, p21^Cip1^, p16^INK4a^, trichoplein, and glyceraldehyde-3-phosphate dehydrogenase (GAPDH). As controls of immunoblotting with p27^Kip1^ or p53/p21^Cip1^, RPE1 cells were subjected to 72 h serum starvation or UV irradiation (10 J/m^2^, 24 h), respectively. These are original data for this review

We have made observations suggesting that depletion of trichoplein in cycling RPE1 cells resulted not only in cell cycle arrest at the G0/G1 phase as reported previously [64] but also in a marked increase in p27^Kip1^ protein level, whereas p53 and p21^Cip1^ levels decreased and p16^INK4a^ level almost unchanged (Fig. 5). Although Doxsey and coworkers reported that p38-p53-p21^Cip1^ pathway-dependent G1/S arrest is induced after depletion of several centrosome-associated proteins, some of which are implicated in primary cilia [108], it appears not to be the case for trichoplein-induced ciliary structural checkpoint for cell cycle re-entry (Fig. 5). p27^Kip1^ is one of the most well-studied mammalian CDK inhibitors (CKIs), which is abundant in G0/G1 cells and is down-regulated in proliferating cells and in S/G2 phase cells [109, 110]. p27^Kip1^ acts in G0 and early G1 to inhibit G1 cyclin/CDK2 complexes, with the primary target being cyclin E/CDK2 [109, 110]. The protein level of p27^Kip1^ is mainly regulated by proteasomal degradation with three ubiquitin ligases [71, 110–113]. Among them, SCF^Skp2^ ubiquitylates and degrades p27^Kip1^ in late G1/S/G2 phases [71, 110], whereas KPC1 and Pirh2 function at early time points of cell cycle entry [71, 111–113]. It is of interest in the future to examine whether trichoplein loss induces p27^Kip1^ accumulation through the inhibition of these three ubiquitin ligases.

## A possible involvement of ciliary length signal in regulation of cell cycle

As described above, in flagellar shortening of *Chlamydomonas*, it is becoming clear that a length signal can be transmitted from cilia to the cytoplasm to control cargo loading, regulating cilium length [34, 82–85, 114, 115] (Fig. 4). In addition, Pan’s group found that ciliary shortening itself is able to induce a phosphorylation signaling cascade [101]. Of note, this ciliary length signal originates at cilia but not at membrane receptors or channels, suggesting that cilia transmit two types of signals to cytoplasm, in which one is emanated from the membrane, the other is from the axoneme. Since it is reported that cells depleted of Nde1 have longer cilia and a delay in cell cycle re-entry that correlates with ciliary length [65], it is plausible that a similar system to transmit a ciliary length information from a cilium itself may also exist in mammalian cells. Thus, it is tempting to speculate that the information of axonemal length could be transmitted into the cytosol even in mammalian cells similar to *Chlamydomonas*, and this signal might regulate key molecules to regulate G0/G1-S progression such as p27^Kip1^, thereby primary cilia may function as a structural checkpoint in cell cycle re-entry (Fig. 4). The strength of this axonemal length signal to suppress cell cycle re-entry may be proportional to axonemal length, which is consistent with a model of a coordination of ciliary length and cell cycle progression by Nde1 [26, 35, 65] (Fig. 4).

## Cellular context-dependent linkage between cilia and cell cycle

As described above, in most cells, primary cilia start to disassemble when cells re-enter the cell cycle, whereas there are some exceptional cases of cells that retain cilia during cell proliferation [8, 13, 26, 38, 39, 107, 116]. For example, Riparbelli et al. demonstrated that cilia assemble and persist during two meiotic divisions in *Drosophila* spermatocytes, raising the possibility that the cilium retention during the cell division may occur diverse organisms and cell types [116].

During embryogenesis and postnatal neurogenesis, cells of the neuroepithelium divide rapidly but at the same time sense a Hedgehog morphogen gradient through primary cilia to adopt a given neuronal fate [8, 117–122]. In this cell context, a primary cilium plays a key role in transducing Hedgehog (and possibly other) signals that maintain neuroepithelial cells in a proliferative state [120, 123, 124]. Das and Storey have elegantly observed the process of neuronal differentiation from proliferating neuroepithelial cells [124]. They demonstrated that in the process of transformation into postmitotic neurons, N-cadherin down-regulation is required for cilia disassembly, centrosome release from the apical surface, as well as for final abscission of apical membrane, which ensures down-regulation of Hedgehog signaling and cell cycle exit as defined by p27^Kip1^ expression [124]. Thus, primary cilia are necessary for neuroepithelial cells to receive a growth signal and proliferate, which may be seemingly contradictory to the hypothesis that primary cilia suppress the cell cycle progression. In this regard, Sung and coworkers made a meaningful observation [36]. As mentioned earlier, they showed that IGF-1 transmits its proliferative signals primarily through the ciliary disassembly to release the ciliary brake for the G1/S transition. Since compromising the formation of cilia in IFT mutant cells eliminates their ability to proliferate in response to IGF-1, IGF-1 and the downstream signaling cascade require primary cilia to couple cilia disassembly with cell cycle progression [36]. It is therefore possible that Hedgehog signal might also maintain the proliferative state of neuroepithelial cells through cilia in a similar way to the IGF-1 signal, in which the transient cilia absorption occurs to abolish the ciliary restrictions on the cell cycle progression. However, the permanent loss of cilia might inhibit the transmission of Hedgehog proliferation signal and induce their terminal differentiation into postmitotic neurons. Sung and colleagues also described that non-ciliated IFT mutant cells, which are unable to respond IGF-1, enter S phase in response to serum at a ~2-fold higher rate relative to their wild-type, ciliated counterparts [36]. They suggested that these findings might help to address why cilia have opposing effects on cell growth, depending on the context [36].

In cancer, the relationship between cilia and tumor proliferation is reported to be complex and controversial [13, 15, 17, 18, 20, 39]. Some studies demonstrated the correlation between loss of cilia and tumor development and proliferation but others failed to support this evidence [13, 14, 18, 19, 35, 125]. Han et al. reported a concept to consider cilia in tumor biology, in which genetic ablation of primary cilia blocked medulloblastoma formation in conditions under which this tumor is driven by a constitutively active Smoothened protein, an upstream activator of Hedgehog signaling, whereas removal of cilia is required for medulloblastoma growth by a constitutively active glioma-associated oncogene family zinc finger-2 (Gli2), a downstream transcription factor [16]. Reiter et al. [126] also found that primary cilia can either mediate or suppress Hedgehog pathway-dependent tumor formation in basal cell carcinomas. Thus, primary cilia are clearly either driving or inhibiting tumorigenesis, depending on the initiating oncogenic event [16, 126]. It is, therefore, conceivable that the cell origin of tumors, genetic background of tumors, and impaired signaling in tumors must be taken into consideration when we examine the relationship between cilia and tumor proliferation.

## Extra-ciliary functions of ciliary proteins

It has been uncovered that cilia proteins are present at non-cilia sites, where they exert cilia-independent functions (see for review [127]). For example, overexpression of IFT88 prevents G1/S transition in non-ciliated cells by inhibiting the interactions of Che-1 with Rb, freeing Rb to repress E2F1 [128]. IFT88 is also implicated in spindle orientation in mitosis [129, 130] as well as epithelial cell migration [131]. Since cilia proteins have been increasingly found at various cellular organelles and structures that collectively perform diverse cellular functions [127], we must carefully take it into account when we evaluate whether primary cilia directly regulate cell cycle progression. We and others used cilia-depleted conditions by means of the knockdown of IFT88, IFT20, or Talpid3 to observe the cilia-dependent effects of ciliary proteins on cell cycle progression [35, 64, 65, 98]. Although we and others indeed confirmed cautiously that IFT88, IFT20, or Talpid3 knockdown alone had only marginal effects on cell cycle in the experimental conditions used [35, 64, 65, 98], it is very important to keep in mind the extra-ciliary effects of ciliary proteins when we seek to more precisely determine the relationship between primary cilia and cell cycle in the future.

## Conclusions

In protozoa, cilia emerged and developed as sensory and motor organelles. In complex multicellular organisms like humans, cells have evolved to utilize primary cilia as a means to orchestrate proliferation and differentiation, setting in which the reciprocal regulation of primary cilia and the cell cycle has a substantial role. With the exception of some cells ciliated during cell proliferation, it is becoming evident that the persistent existence of primary cilia per se prevents cell cycle re-entry and proliferation, which might to be potentially relevant to well-known observations that tumor cells frequently lose their primary cilia. The further clarification of the link between primary cilia and cell cycle will contribute to a more precise understanding of the pathology of cilia-related disease including cancer as well as the discovery of new targets of therapeutic interventions.

## Abbreviations

APC: anaphase-promoting complex
CDK: cyclin-dependent kinase
CRL3s: Cul3-RING E3 ligases
CV: ciliary vesicles
DAP: distal appendage
HDAC6: histone deacetylase 6
IFT: intraflagellar transport
IGF-1: insulin-like growth factor-1
Inpp5e: inositol polyphosphate 5-phosphatase E
MARK4: microtubule-associated protein/microtubule affinity regulating kinase 4
MTOC: microtubule organizing center
Nde1: nuclear distribution gene E homologue 1
Ofd1: Orofaciodigital syndrome 1/Oral-facial-digital syndrome 1
PI: phosphatidylinositol
SCF: Skp1/Cullin1/F-box protein
TTBK2: Tau tubulin kinase 2
